# “Plurethosome” as Vesicular System for Cutaneous Administration of Mangiferin: Formulative Study and 3D Skin Tissue Evaluation

**DOI:** 10.3390/pharmaceutics13081124

**Published:** 2021-07-23

**Authors:** Maddalena Sguizzato, Francesca Ferrara, Paolo Mariani, Alessia Pepe, Rita Cortesi, Nicolas Huang, Fanny Simelière, Paola Boldrini, Anna Baldisserotto, Giuseppe Valacchi, Elisabetta Esposito

**Affiliations:** 1 Department of Chemical and Pharmaceutical Sciences, University of Ferrara, I-44121 Ferrara, Italy; sgzmdl@unife.it (M.S.); crt@unife.it (R.C.); 2 Department of Neurosciences and Rehabilitation, University of Ferrara, I-44121 Ferrara, Italy; frrfnc3@unife.it; 3 Department of Life and Environmental Sciences, Polytechnic University of Marche, I-60131 Ancona, Italy; p.mariani@staff.univpm.it (P.M.); a.pepe@pm.univpm.it (A.P.); 4 Institut Galien Paris-Saclay, CNRS, Université Paris-Saclay, 92296 Châtenay-Malabry, France; nicolas.huang@universite-paris-saclay.fr (N.H.); fanny.simeliere@universite-paris-saclay.fr (F.S.); 5 Center of Electron Microscopy, University of Ferrara, I-44121 Ferrara, Italy; bdp@unife.it; 6 Department of Life Sciences and Biotechnology, University of Ferrara, I-44121 Ferrara, Italy; bldnna@unife.it; 7 Animal Science Department, NC Research Campus, Plants for Human Health Institute, NC State University, Kannapolis, NC 28081, USA; 8 Department of Food and Nutrition, Kyung Hee University, Seoul 02447, Korea

**Keywords:** phosphatidylcholine, poloxamer, mangiferin, in vitro diffusion, antioxidant

## Abstract

Human skin is dramatically exposed to toxic pollutants such as ozone. To counteract the skin disorders induced by the air pollution, natural antioxidants such as mangiferin could be employed. A formulative study for the development of vesicular systems for mangiferin based on phosphatidylcholine and the block copolymer pluronic is described. Plurethosomes were designed for mangiferin transdermal administration and compared to ethosome and transethosome. Particularly, the effect of vesicle composition was investigated on size distribution, inner and outer morphology by photon correlation spectroscopy, small angle X-ray diffraction, and transmission electron microscopy. The potential of selected formulations as vehicles for mangiferin was studied, evaluating encapsulation efficiency and in vitro diffusion parameters by Franz cells. The mangiferin antioxidant capacity was verified by the 2,2-diphenyl-1-picrylhydrazyl assay. Vesicle size spanned between 200 and 550 nm, being influenced by phosphatidylcholine concentration and by the presence of polysorbate or pluronic. The vesicle supramolecular structure was multilamellar in the case of ethosome or plurethosome and unilamellar in the case of transethosome. A linear diffusion of mangiferin in the case of ethosome and transethosomes and a biphasic profile in the case of plurethosomes indicated the capability of multilamellar vesicles to retain the drug more efficaciously than the unilamellar ones. The antioxidant and anti-inflammatory potential effect of mangiferin against pollutants was evaluated on 3D human skin models exposed to O_3_. The protective effect exerted by plurethosomes and transethosomes suggests their possible application to enhance the cutaneous antioxidant defense status.

## 1. Introduction

In the 21st century, one of the major environmental risks to human health is represented by air pollution, causing a reduction of life expectancy by almost 2 years on worldwide average [1]. Indeed, it has been demonstrated that many respiratory diseases, cardiovascular dysfunctions, as well as skin pathologies can be induced by toxic substances produced by humans and accidentally delivered into the atmosphere [2,3,4]. These volatile organic compounds contribute to secondary pollutant formation, such as the tropospheric ozone O_3_ [5]. O_3_ can be considered as one of the most toxic pollutants present within the air. The noxious effect of O_3_ on human skin has been thoroughly investigated in the past decades, demonstrating its interaction with *stratum corneum* macromolecules (i.e., lipids, proteins) that leads to the production of reactive oxygen species (ROS) and secondary mediators, such as the reactive aldehyde 4-hydroxy-nonenal (4HNE) [6]. These oxidative stress mediators can spread the oxidative damage throughout the skin layers and eventually induce the activation of inflammatory pathways, resulting in the release of inflammatory cytokines as interleukins (ox-inflammation) [7,8] that are involved in the development and/or exacerbation of conditions such as atopic dermatitis, skin photoaging, photo carcinogenesis, DNA damage, and cell death [9]. In this respect, in order to treat dermatological diseases or to prevent the development of skin pathologies by air pollution, efficacious strategies are needed [10]. A successful approach based on the cutaneous application of natural antioxidant molecules has been well investigated in the last decades. Among them, mangiferin (MG), a natural glucosyl xanthone found in both mango and papaya, has been recently proposed [11]. MG exerts several pharmacological activities, such as hepatoprotective, anticarcinogenic, antidiabetic, and antiviral action against herpes simplex virus and polio virus [12,13,14,15]. Notably, due to its antioxidant, antiapoptotic, and antiinflammatory potential, MG has been proposed in the treatment of cutaneous diseases such as contact dermatitis and psoriasis [11,16]. Notwithstanding its therapeutic potential, MG is characterized by scarce water solubility and low bioavailability, requiring advanced drug delivery systems for its administration [17,18]. In this respect, vesicular nanosystems could represent a smart technological strategy for MG application and delivery through the skin. Thanks to the use of biocompatible components such as phosphatidylcholine (PC), lipid-based nanovesicular systems are suitable for skin administration, especially to achieve the transdermal delivery of drugs [19,20,21]. Particularly, ethosomes are vesicular multilamellar nanosystems mainly based on PC and ethanol (20–45%) suitable for drug solubilization and delivery through the skin, due to the dual effect of PC and ethanol [22,23]. Ethanol presence is crucial, since on one hand, it confers softness and malleability to the PC vesicle, while on the other, it exerts a penetration enhancer effect, promoting the ethosome passage through the skin. The addition of surfactants to PC in ethosome composition has led to the development of transethosome, a second generation of ethosome, which is characterized by even more flexible vesicles with major transdermal potential [18,24,25]. The uptake of ethosome and transethosome within keratinocytes has been recently demonstrated by transmission electron microscopy analyses, confirming their penetration enhancer ability and their capability to deliver drugs within cells [18,25]. Since the oxidative stress mediators can alter the expression of *stratum corneum* lipid components, the cutaneous administration of lipid vesicular nanosystems appears particularly interesting in the treatment of skin pathologies, offering the possibility to restore the lipid depletion caused by environmental pollutants [26]. Recently, we demonstrated the potential of MG entrapped in ethosome and transethosome in the treatment of cutaneous ox-inflammatory damage induced by cigarette smoke [18]. In the present investigation, PC-based vesicular nanosystems were further investigated, including in their composition the non-ionic triblock copolymer Pluronic F127 (Poloxamer 407). Pluronics are constituted of a hydrophobic polypropylene oxide group sandwiched between hydrophilic polyethylene oxide moieties [27]. Depending on its concentration, this special kind of surfactants can self-associate in micelles or form highly ordered aggregates [28]. The choice of investigating the so-called “plurethosomes”, based on pluronic in mixture with PC and ethanol, is related to the copolymer capability to interact with lipid bilayers. Indeed, PPO blocks in pluronics can disturb the bilayer lipid packing of the cellular membrane, thus increasing the permeation of associated drugs [29]. Thus, a comparative study was conducted, investigating the effect of the non-ionic surfactant polysorbate 80 and Pluronic F127 on vesicular nanosystem preparation. After thorough characterization, selected vesicular nanosystems were studied as MG transdermal delivery systems, evaluating their antioxidant and anti-inflammatory potential in 3D human skin tissues exposed to O_3_ to induce ox-inflammatory damage. 

## 2. Materials and Methods 

### 2.1. Materials

Mangiferin (MG), the copolymer poly(ethylene glycol)-block-poly(propylene glycol)-block-poly(ethylene glycol) poloxamer 407 (Pluronic F127, F127) (PEO98-POP67-PEO98), and polysorbate 80 (T80) were purchased from Sigma-Aldrich (St Louis, MO, USA). The soybean lecithin (PC) (90% phosphatidylcholine) was Epikuron 200 from Lucas Meyer (Hamburg, Germany). Nylon membranes were purchased from Merck (Milan, Italy). Solvents were of HPLC grade, and all other chemicals were of analytical grade.

### 2.2. Vesicular Nanosystem Preparation 

Vesicular nanosystems were prepared by the addition of bidistilled water into an ethanolic solution of PC (90% *w*/*v*) [18]. Namely, water was slowly added to the ethanol phase up to a final water/ethanol ratio of 20:80 or 70:30 (*v*/*v*), under magnetic stirring at 750 rpm (IKA RCT basic, IKA^®^-Werke GmbH & Co. KG, Staufen, Germany) for 30 min at room temperature. In the case of transethosome or plurethosome preparation, T80 or F127 were respectively solubilized in the PC ethanol solution before water addition. MG-containing nanosystems were prepared, solubilizing the drug (0.8 mg/mL) alternatively in the PC, PC/T80, or PC/F127 ethanol solution before adding water. The macroscopic appearance of vesicular nanosystems stored at 25 °C in the light was investigated during 3 months. Particularly, possible phase separation or sedimentation phenomena, as well as color and transparency changes were evaluated.

### 2.3. Photon Correlation Spectroscopy (PCS)

Size analysis of vesicular nanosystems was performed by a Zetasizer Nano-S90 (Malvern Instr., Malvern, UK) equipped with a 5 mW helium neon laser and a wavelength output of 633 nm. Measurements were performed at 25 °C, 90° angle, and a run time of at least 180 s. After sample dilution with bidistilled water (1:20 *v/v* ratio), data were analyzed using the “CONTIN” method [30]. Measurements were taken 1 and 90 days from vesicular nanosystem production and repeated thrice on different samples.

### 2.4. Transmission Electron Microscopy (TEM)

Samples for TEM analyses were negatively stained, depositing a sample drop on a TEM screen covered with a Formvar film (Media System Lab S.r.l., Macherio, MB, Italy). The excess drop was removed after 1 min from the screen with filter paper to keep a light veil of sample on the supporting substrate. A drop of 2% phosphotungstic acid was placed on the screen for 1 min and then removed with filter paper to surround the nanosystems deposited on the screen and adhere to their surface. Then, the screen was observed with a ZEISS EM 910 transmission electron microscope (Carl Zeiss Microscopy, GmbH, Munich, Germany).

### 2.5. X-ray Scattering

Small angle and wide-angle X-ray scattering (SAXS and WAXS) experiments were conducted at the I22 beamline of Diamond Light Source (Harwell, UK). The experiment exploited the mail-in service. The 3m camera anisotropic SAXS/WAXS I22 setup and a Pilatus P3-2M (Silicon hybrid pixel detector, DECTRIS) detector with a pixel size of 172 µm^2^ were used. The final investigated Q-range was 0.4–3.0 nm^−1^ for SAXS and 3.0–50 nm^−1^ for WAXS. Samples were prepared in a capillary rack, each capillary having a diameter of 1 mm. Two-dimensional (2D) data were corrected for background, detector efficiency, and sample transmission and then radially averaged to derive I(Q) vs. Q curves [31].

### 2.6. Mangiferin (MG) Content of Vesicular Nanosystems

The entrapment capacity (EC) of MG in vesicular nanosystems was determined after production. Five hundred microliters of each sample were loaded in a centrifugal filter (Microcon centrifugal filter unit YM-10 membrane, NMWCO 10 kDa, Sigma-Aldrich, St. Louis, MO, USA) and ultra-centrifuged (Spectrafuge™ 24D Digital Microcentrifuge, Woodbridge, NJ, USA) at 4000 rpm for 15 min. Afterwards, a 100 μL aliquot of supernatant (in the upper section of the filter unit) was diluted with ethanol (1:10, *v*/*v*) and maintained under magnetic stirring for 30 min [18]. After filtration of the solution by nylon syringe filters (0.22 μm pores), MG amount was analyzed by HPLC as below reported. The EC was determined as follows:

EC = MG/T_MG_ × 100 (1)

where MG is the amount of drug measured by HPLC and T_MG_ is the total amount of MG employed for nanosystem production. The MG amount in the dispersing phase (in the lower section of the filter unit) was analyzed by HPLC as below reported. The MG recovery was calculated considering the amount of MG associated to the vesicles plus the amount of MG found in the dispersing phase:

Recovery = MG_vesicles_ + MG_dispersing phase_/T_MG_ × 100 (2)

where MG_vesicles_ is the amount of drug in the upper section of the filter unit, MG_dispersing phase_ is the amount of drug in the upper section of the filter unit, and T_MG_ is the total amount of MG employed for nanosystem production.

### 2.7. In Vitro Diffusion Experiments

In vitro MG diffusion was investigated by Franz cells assembled with nylon membranes (2 cm diameter, pore size 0.2 µm) (_Vetrotecnica_, Padova, Italy) [18]. Franz cells were set up of a lower receptor and an upper donor compartment separated by a membrane, whose exposed surface area was 0.78 cm^2^ (1 cm diameter orifice). Before starting the experiment, the membranes were hydrated with the receiving phase for 1 h. Five milliliters of ethanol/water (50:50, *v*/*v*) were poured in the lower section, stirred at 500 rpm by a magnetic bar, and thermostated at 32 ± 1 °C during all the experiments [32]. Approximately 1 g of MG-containing forms (MG 0.8 mg/mL) was placed in the donor compartment and sealed to avoid evaporation. At predetermined time intervals (0.5–6 h), 200 µL of receiving phase were withdrawn, replaced with an equal volume, and analyzed for MG content by HPLC. The MG concentrations were determined six times in independent experiments, and the mean values ± standard deviations were calculated. The accumulation curves were obtained plotting mean values as a function of time. The fluxes were extrapolated from the linear portion of the curves, considering the slopes of the regression line (angular coefficient). At last, the diffusion coefficients were calculated according to Equation (2).

D = F/[MG](3)

where D is the diffusion coefficient, F is the flux, and [MG] is the MG concentration in the analyzed form, expressed in mg/mL. The concentrations of MG retained in the vehicles (Rv) and in the membrane (Rm) at the end of the diffusion experiments were also quantified [33]. Namely, 100 μL aliquots of vehicle were withdrawn, diluted with ethanol (1:10, *v*/*v*), maintained under magnetic stirring for 30 min, and analyzed by HPLC after filtration. The membranes were removed from the Franz diffusion cell, gently dabbed with paper to remove excess of vehicle, placed in 5 mL of ethanol, underwent sonication for 30 min, filtered through a nylon syringe filter (pore size 0.22 μm), and analyzed by HPLC. To evaluate the affinity of MG for the membrane and the influence of the vehicle, Rm/Q6 and Rv/Q6 ratios were calculated, considering the total amount of MG diffused after 6 h (Q6).

### 2.8. HPLC Analysis

For HPLC analyses, a two-plunger alternative pump (Agilent Technologies 1200 series, Santa Clara, CA, USA), a UV detector operating at 254 nm, and a 7125 Rheodyne injection valve with a 50 μL loop have been employed. Analyses were conducted eluting a stainless-steel C-18 reverse-phase column (15 × 0.46 cm) packed with 5 μm particles (Platinum C18, Apex Scientific, Maynooth, Co. Kildare, W23 R1H2, Ireland) with a mobile phase containing methanol/water 60:40 *v*/*v*, pH 4.0 at a flow rate of 1 mL/min. 

### 2.9. Antioxidant Activity (2,2-diphenyl-1-picrylhydrazyl Assay)

The antioxidant activity of MG-containing vesicular nanosystems was tested and compared to a 30:70 *v/v* ethanol solution of the drug. In particular, for the evaluation of the DPPH radical scavenging activity, a previously described [18] modified protocol was used that is suitable for phenolic compounds and for a rapid in vitro evaluation of antioxidant capacity. Briefly, the conversion of the stable free radical DPPH into 1, 1-diphenyl-2-picrylylhydrazide by a hydrogen-donating antioxidant involves a colorimetric variation of the solution from deep purple to yellow (the reaction between the test substance and the radical), which is monitored by reading at 517 nm with a UV-Vis spectrophotometer (range 190–1100 nm) (UV-31 SCAN ONDA, Sinergica, Milano, Italy). The radical inhibition percentage is calculated using the following equation:

DPPH radical-scavenging capacity (%) = [1 − (A_1_ − A_2_)/A_0_] × 100% (4)

where A_0_ is the absorbance of the control (without sample), A_1_ is the absorbance in the presence of the sample, and A_2_ is the absorbance without DPPH.

Seven hundred and fifty microliters of each sample diluted in ethanol at different concentrations were added to the DPPH ethanolic solution (1.5 mL), and the absorbance was measured by UV-Vis spectrophotometer. The IC_50_ values were calculated from the results, and determined by regression analysis of the results obtained at different sample concentrations. The values obtained were the results of at least three different experiments and expressed as the mean value (µg/mL) ± the standard deviation.

### 2.10. Rheological Measurements

Rheological measurements of vesicular nanosystems were performed with an AR-G2 controlled-stress rotational rheometer (TA Instruments, New Castle, DE, USA) [34]. The geometry used was an aluminum cone-plate with a diameter of 40 mm, an angle of 1°, and a truncation of 28 µm, which was equipped with a solvent trap to prevent solvent evaporation during the experiments. The viscoelastic properties of the samples (elastic modulus G′ and viscous modulus G″) were assessed in oscillation mode. Oscillation frequency was set at 1 Hz, and the deformations applied were all in the linear regime. Temperature ramps from 5 to 50 °C were obtained at a temperature rate of 1 °C/min and were controlled by a Peltier plate. Before starting the experiments, a 2 min conditioning time at 5 °C was applied. Measurements were performed thrice at least for each sample to ensure reproducibility.

### 2.11. Culture and Exposure to Ozone of EpiDerm 3D Skin Models

EpiDerm (EPI-200) skin model samples were obtained from MatTek corporation (Ashland, MA, USA) and transferred into 6-well plates prefilled with 1 mL of MatTek assay medium upon arrival, following the manufacturer’s protocol [35]. After 24 h of recovery in an incubator (5% CO_2_, 37 °C), 1 mL of fresh new medium was added to each well to topically pretreat the tissues with 35 μL of vesicular nanosystems for 24 h. Afterwards, vesicular nanosystems were placed in a plexiglass box connected to an ozone (O_3_) generator equipped with a detector able to monitor the O_3_ concentration and exposed to 0.4 ppm of O_3_ for 4 h, as previously described [35]. Inserts and media were collected directly after exposure (0 h) or 24 h post-exposure [36]. Untreated tissues exposed to O_3_ at the same conditions were used as reference, while untreated tissues exposed to filtered air were used as control (CTRL). 

### 2.12. Immunohistochemistry for 4HNE

Sections of EpiDerm skin model were previously fixed in formalin and then embedded in paraffin (formalin-fixed paraffin-embedded, FFPE) and finally subjected to immunofluorescence staining for 4-hydroxy-nonenal (4HNE). Briefly, 4 μm sections were deparaffinized, rehydrated, and incubated in a 10 mM citrate buffer pH 6 (AP-9003-500, Thermo Fisher Scientific, Waltham, MA, USA) at 90 °C for 10 min in a 500-watt microwave for the antigen retrieval step. After cooling for 30 min at RT, sections were firstly washed twice in phosphate buffer saline (PBS); then, they were blocked for 45 min in PBS containing 2% bovine serum albumin (Biorad, Hercules, CA, USA) and further incubated overnight at 4 °C with 4-HNE antibody diluted 1:400 in PBS and BSA 0.2% (AB46545, Abcam, Burlington, MA, USA). Then, tissue sections were washed 3 times in PBS before incubation with secondary antibody Goat Anti-rabbit IgG H&L (Alexa Fluor^®^ 488; A-11008, ThermoFisher Scientific, Cambridge, UK) (1:1000 in PBS, BSA 0.2%) for 1 h at 25 °C. After washing in PBS, nuclei were stained with a diamidino-2-phenylindole dye DAPI (Tocris Biosciences, Bristol, UK) 300 mM solution for 5 min, then washed again in PBS. Coverslips were subsequently mounted onto glass slides using the Permafluor aqueous mounting medium (ThermoScientificTM, Minneapolis, MN, USA) and consequently imaged via fluorescence on a Nikon Microphot FXA microscope (Nikon Instruments, Amsterdam, The Netherlands) equipped at 40× magnifications. Fluorescence staining intensity was quantified using Imagej software [37].

### 2.13. Detection of IL-1β Using ELISA Assays

After collecting the tissues media at the two different time-points upon O_3_ exposure (0 and 24 h), IL-1β levels were measured by using the Human IL-1 beta/IL-1F2 DuoSet ELISA kit (R&D System cat DY201, Minneapolis, MN, USA), according to the manufacturer’s protocol. IL-1β levels were adjusted for media (pg/mL) and expressed as arbitrary units (AU%). Gen5 software (BioTek, USA) was used for the detection [35].

## 3. Results and Discussion

### 3.1. Preparation of Vesicular Nanosystems

In a previous study, we have demonstrated the possibility of solubilizing and delivering MG in biocompatible vesicular nanosystems containing high amounts of ethanol, namely ethosome and transethosome [18]. Particularly, ethosomes were based on PC 0.9% and ethanol 30%, while in the case of transethosomes, the composition was enriched with the non-ionic surfactant T80 (0.3%) selected by a formulative study. The surface activity and low toxicity of non-ionic surfactants make them particularly suitable as solubilizers and stabilizers for poorly soluble drugs [28]. A very special kind of non-ionic surfactants is typified by the pluronic copolymers. Their composition is based on a central polyoxypropylene block surrounded by two hydrophilic polyoxyethylene blocks, and it confers to pluronics a surfactant character suitable for many pharmaceutical applications [27,28,29]. In the present study, we conducted a preformulative investigation considering the influence of vesicular nanosystem composition on their size distribution and morphology. Particularly, the presence and concentration of PC, T80, and F127 was evaluated. Notably, the addition of F127 to ethosome composition resulted in the production of vesicular nanosystems named “plurethosome”, being mainly composed of F127. Table 1 reports the composition of ethosomes, transethosomes, and plurethosomes. Ethosomes were produced using PC concentration ranging from 0.6 to 2.7% (P1, P5, P7, P15), while transethosomes were based on PC 0.9, 1.8, or 2.7% with the addition of T80 0.3% (P6, P12, P16), the water amount was 80%, apart from P5, P6, P15, and P16, where it was 70%. Ethosome macroscopic appearance was translucent or milky, as a function of PC concentration. PC 1.8% resulted in temporary homogeneous ethosomes (P7), undergoing phase separation within 15 days, while a further increase in PC concentration up to 2.7% led to an unstable dispersion (P15), which was subject to sedimentation and phase separation within 2 days (Table 2). Conversely, in transethosomes, the presence of T80 conferred a translucent aspect and allowed stabilizing the dispersions containing the highest amount of PC (P12 and P16), preventing phase separation. Plurethosome production was achieved by solubilizing F127 (0.3–1.6%) into PC ethanol solution (30 or 90 mg/mL) before water addition. In the case of P2–P4, based on PC 0.6%, obtained using 20% of a PC ethanol solution (30 mg/mL), the addition of F127 0.3% did not lead to macroscopic changes (P2), while an increase of F127 amount (P3, P4) changed the macroscopic appearance and led to phase separation of the dispersions after few days. Probably, F127 disorganized the vesicle assembly at low PC concentration, forming PC/F127 mixed micelles. To achieve more stable dispersions, F127 was solubilized in 3-fold concentrated PC solution (90 mg/mL) before water addition, resulting in a final PC concentration of 1.8% (P8–P11, P14). This procedure gave rise to milky plurethosome dispersions almost devoid from phase separation phenomena, apart from P8 and P14, containing the lowest and the highest amounts of F127 (0.3%), undergoing phase separation respectively 90 or 30 days after preparation. The addition of both T80 and F127 to PC solution (90 mg/mL) resulted in P13, appearing translucent and free from phase separation phenomena. 

### 3.2. Characterization of Vesicular Nanosystems

**Figure 1:**
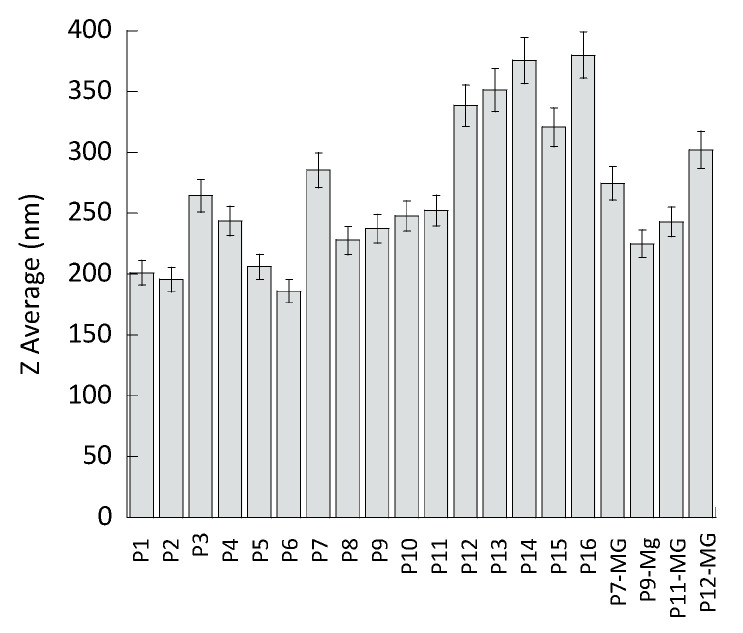
Mean diameter of the indicated formulations. Diameters were measured by PCS and expressed as Z average (nm). Data are the mean of three determinations on different samples ± s.d.

To get information about the influence of composition on size distribution of vesicular nanosystems, mean diameters and dispersity indexes were evaluated by PCS. As reported in Table 2 and depicted in Figure 1, mean diameters, expressed as Z average, ranged between 190 and 550 nm. Ethosome mean diameter was affected by the PC concentration, passing from 201 nm in the case of P1 (PC 0.6%) to 320 nm in the case of P15 (PC 2.7%). In transethosomes, T80 presence slightly decreased the mean diameter in the case of PC 0.9%, while at PC concentrations 1.8% and 2.7%, T80 0.2% and 0.3% led to a notable increase in mean diameter, namely +53 nm and +230 nm in the case of P7 and P16, respectively.

In the case of plurethosomes, F127 0.3–1.2% induced a slight increase in the mean diameter of vesicles constituted of PC 0.6% (P2–P4) or PC 1.8% (P8–P11), whilst a further F127 increase, up to 1.6%, resulted in a 120 nm enlargement of the mean diameter in PC 1.8% vesicles (P14). In the case of P13, containing F127 1.2%, the presence of T80 0.2% led to a 100 nm increase, with respect to the corresponding plurethosome dispersion P11. Thus, as a general rule, the size of vesicles was influenced by PC amount: the higher the PC concentration, the higher the mean diameter. Notably, considering vesicles based on the same amount of PC (1.8%), in the case of plurethosome, F127 (0.3–1.2%) led to a decrease of mean diameter, whilst in the case of transethosome, T80 (0.2–0.3%) increased mean diameter with respect to ethosome. Dispersity indexes were always below 0.3, suggesting monomodal size distributions, the highest values were obtained in the case of P15 and P16, which were produced with PC 2.7%.

**Figure 2:**
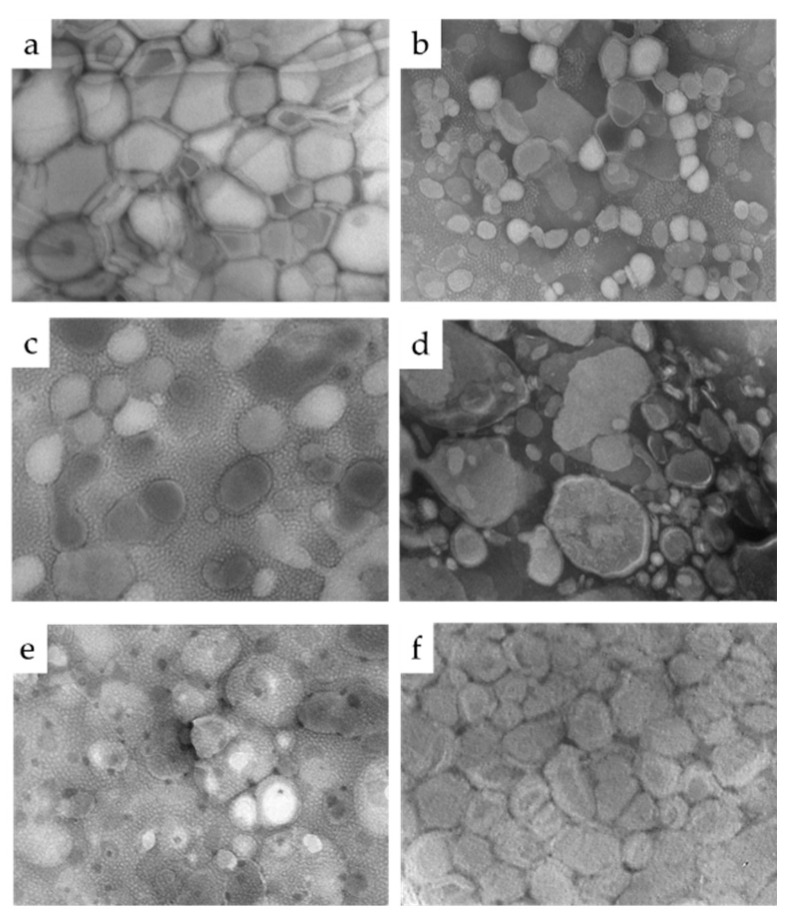
Transmission electron microscopy images (TEM) of P7 (**a**), P9 (**b**), P11 (**c**), P12 (**d**), P13 (**e**), and P16 (**f**). Bar corresponds to 200 nm (**a**,**c**,**d**) or 500 nm (**b**,**e**,**f**).

In order to better investigate the influence of T80 and F127 on morphology and inner structure of PC vesicular nanosystems, TEM analyses and SAXS measurements were conducted. Particularly, P7, P9, P11, P12, P13, and P16 were selected as representative examples of ethosome, transethosome, and plurethosome. As appreciable from the TEM images reported in Figure 2, ethosome (P7), transethosome (P12, P16), and plurethosome (P9, P11, P13) vesicles appear roundish, while their mean diameters correlate well with Z average values measured by PCS.

**Figure 3:**
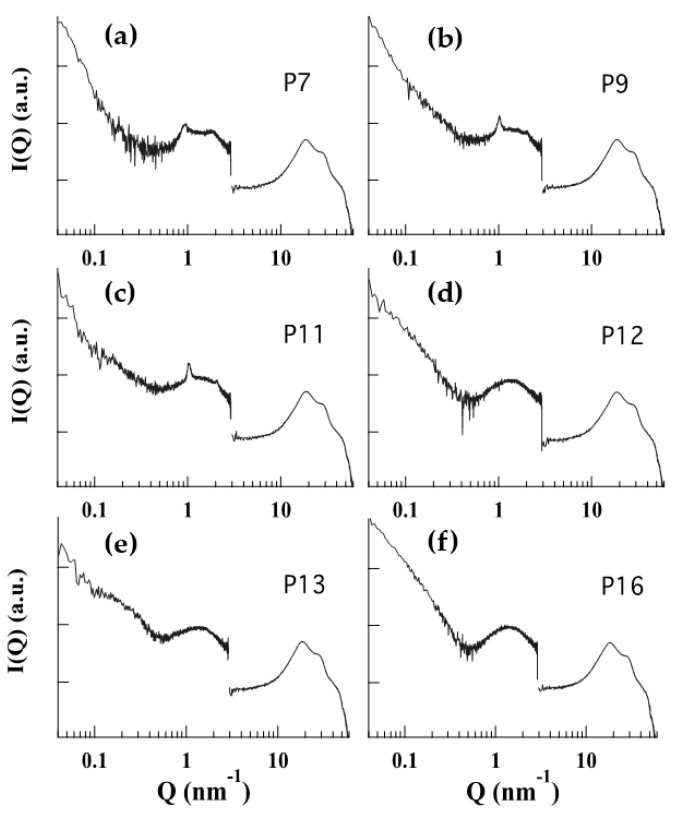
SAXS/WAXS profiles observed at 35 °C. P7 (**a**), P9 (**b**), P11 (**c**), P12 (**d**), P13 (**e**), and P16 (**f**).

Scattering profiles and diffraction parameters are reported in Figure 3 and Table 3, respectively. While the WAXS region (curves in the Q-range from 3.0 to 50 nm^−1^) is very similar for all samples, SAXS profiles show two different typologies. In the case of P12, P13 and P16, a typical bilayer form factor scattering pattern (characterized by the broad band centered at about 1.4 nm^−1^) is detected, while the SAXS profiles for P7, P9, and P11 show the presence of two low-intensity Bragg peaks superposed to the bilayer form factor. The two different profiles indicate different structural properties of the vesicular nanosystems: at one side, the bilayer form factor scattering pattern is indicative of the occurrence of unilamellar vesicles; at the other side, the presence of superposed peaks, with spacing in a ratio of 1:2, reveals the formation of multilamellar vescicles [34,38]. Therefore, in ethosome, PC 1.8% (P7) led to the formation of multilamellar vesicles, as found for PC 0.9% [34]. In good agreement with previous results, the lamellar repeat distance, as determined by the peak spacings, was 6.76 nm (see Table 3). The addition of pluronic F127 (as in P9 and P11) did not modify the multilamellar structure, even if the observed increase of the Bragg peak intensity together with the reduction of the lamellar repeat distance suggest that a more packed organization of the lamellae was induced. Indeed, F127 in water is known to form spherical polymeric micelles, with a PPO hydrophobic core and a hydrophilic PEO screen, with each F127 molecule bent over to assume a “V”-shaped disposition [39]. In the presence of PC, molecular dynamic simulation demonstrated that pluronics (P85 and F68, in particular) strongly interact with lipid bilayers [40]. In particular, it has been shown that the hydrophilic blocks interact with lipid headgroups and then dissolve at the lipid/water interface, whereas hydrophobic blocks insert into the central hydrophobic region of the bilayer (see also Figure S2). It is likely that also F127 behaves in the same way, with the PEO moyeties at the lipid interface probably interacting each other, thus stabilizing the multilamellar packing and reducing the lamellar repeat distance. A stabilizing effect of PC vesicles by F127 was previously described by other authors [41,42]. Noticeably, the presence of F127 inside the bilayer did not affect the lipid hydrocarbon liquid-like conformation, as demonstrated by the very similar WAXS profiles observed in the different conditions. In contrast to Pluronic F127, the addition of T80 (as in P12, P13, and P16) appears to destroy the multilamellar organization (or to induce a very disordered positional correlation between adjacent bilayers) even in the presence of F127. This suggests that T80 interacted differently with the surface of PC vesicles compared to the block copolymer structure of Pluronic F127. The insertion of the oleate chain of T80 into the lipid bilayer and a non-uniform swelling of the aqueous compartments dominated by the polar terminal groups of the POE chains may induce the disordering of the lamellar organisation [43]. The differences in lamellar structure of poloxasome versus ethosome was further confirmed by TEM images (Figure S1), showing a multilamellar pattern in the case of P11, which is not detectable in the case of P12. In the case of P13 formulation, where both T80 and F127 are present, the size and diffraction results are more similar to transethosome samples than to plurethosome ones (Table 2 and Table 3), suggesting that the interaction of T80 with PC molecules overbears the affinity with F127, which in turn could self-organize, forming micelles. This latest hypothesis is corroborated by the TEM image in Figure 2e, showing dark spots not detectable in panels c (P11) and d (P12), containing alternatively F127 or T80. In this regard, a TEM image of F127 1.2% solution in Figure S2 shows F127 micelles, appearing as dark spots with a mean diameter around 30 nm, such as the ones present in the P13 sample (Figure 2e). P13 and P16 were not considered in further experiments due to their large size possibly hampering their passage through the stratum corneum skin barrier. Meanwhile, in order to investigate the influence of composition of vesicular nanosystems on MG encapsulation and delivery, P7, P9, P11, and P12 were selected, being characterized by the same amount of PC and the alternative presence of F127 (0.6 and 1.2%) or T80 (0.2%).

### 3.3. Preparation and Characterization of MG-Containing Vesicular Nanosystems

MG-containing ethosomes, transethosomes, and plurethosomes were produced solubilizing the drug in the PC ethanol phase before water addition (Table 1). MG-containing vesicular nanosystems appeared as yellowish homogeneous dispersions, more translucent in the case P12-MG (Table 2). The presence of MG slightly affected the mean diameter of vesicles, ranging between 225 and 332 nm. P7-MG ethosomes underwent sedimentation within 15 days, similarly to the corresponding P7 dispersion, while P9-MG, P11-MG, and P12-MG maintained their homogeneous aspect within 3 months. The amount of MG associated to the vesicles, or to the dispersing aqueous phase was quantified by ultracentrifugation, disaggregation, and HPLC. In all cases, MG recovery (i.e., the amount of MG associated to the vesicles plus the amount of MG found in the dispersing phase) corresponded to the total amount weighed for the vehicle production, confirming that the preparation procedure avoided drug losses on mechanical equipment or degradation due to high temperatures. EC values, i.e., the amount of MG associated to the vesicular phase, were always above 70% (Table 4). The highest EC was obtained in the case of plurethosomes (P9-MG and P11-MG), followed by ethosomes (P7-MG), while the lowest was found in the case of transethosomes P12-MG, suggesting different vesicle permeability. This result agrees with TEM and SAXS findings; indeed, it is probable that in the case of ethosome and plurethosome, the multilamellar supramolecular structure of the vesicle could promote MG solubilization more efficaciously with respect to the unilamellar organization of transethosome [18,38]. Other authors have described the capability of F127 to keep the drugs solubilized and stable within PC vesicles in hybrid liposome particles [44,45].

### 3.4. In Vitro MG Diffusion Kinetics 

To get information about the influence of the different vesicular nanosystem compositions on MG diffusion kinetics, Franz cells associated to nylon membranes were employed. Nylon membranes are commonly employed in Franz cell experiments in order to compare the performances of topical formulations and for quality controls [18,34,46,47,48]. Of course, natural membranes can provide more predictable in vivo drug permeation results with respect to synthetic membranes; nonetheless, their use can involve many inconveniences due to high costs, scarce availability, and ethical limitations. Moreover, the use of human and animal skin may imply variable results, risks of contamination, and special storage conditions. In addition, synthetic membranes such as the nylon ones are usually employed for quality control or to study drug diffusion thanks to their commercial availability, easy storage modalities, and highly reproducible results [46]. 

**Figure 4:**
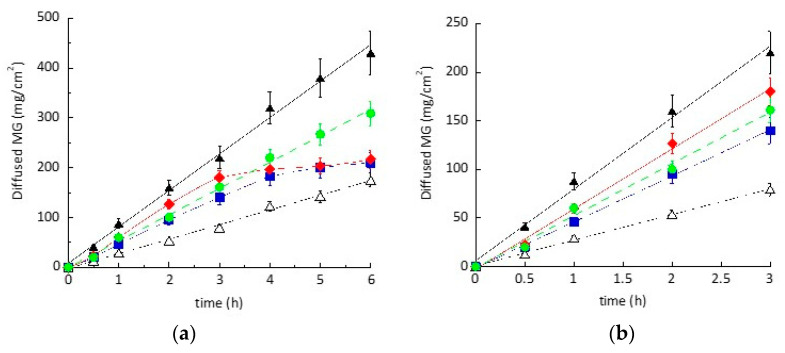
MG diffusion kinetics from Sol-MG (closed triangles), P7-MG (open triangles), P9-MG (green circles), P11-MG (red rhombuses), and P12-MG (blue squares), as determined by Franz cell. Experiments were conducted for 6 h (**a**), with linear profiles within 3 h (**b**). Data are the mean of 6 independent experiments ± s.d.

P7-MG, P9-MG, P11-MG, and P12-MG were investigated and compared to Sol-MG solution. The obtained results are reported in Figure 4. As expected, MG diffusion from ethosome, transethosome, and plurethosome was slower with respect to the simple solution, confirming the vesicle role in controlling drug diffusion. Notably, in the case of ethosome (P7-MG) and transethosome (P9-MG), the MG diffusion profile was linear within 6 h, whilst plurethosomes (P11-MG and P12-MG) displayed a biphasic profile characterized by a linear diffusion up to 3 h, which was followed by a plateau, indicating a slower kinetic. This sustained drug release could be related to a thickened effect of F127 inside the vesicles, as previously reported by other authors [44,45]. 

Table 5 reports F values, reflecting the slope of the linear profiles obtained within 3 h, and D values, calculated considering the MG concentration (Equation (3)). MG diffusion from ethosome was 2-fold slower with respect to transethosome, suggesting the capability of multilamellar structures to restrain MG diffusion with respect to unilamellar ones. In the case of plurethosome, MG diffusion measured in the first 3 h was higher than ethosome, especially in the case of P9-MG, containing a minor F127 amount with respect to P11-MG. 

In order to better investigate the differences in diffusion behavior of MG from the vesicular nanosystems, the amount of drug retained in the donor compartment of the diffusion cell and in the membrane after 6 h was evaluated [33]. In all cases, the amount of MG retained by the nylon membrane (Rm) was negligible with respect to MG diffused through the membrane or residual in the vehicle. The ratio between MG retained by the vehicle with respect to MG diffused after 6 h (Rv/Q6) was higher in the case of P7-MG and P11-MG, confirming that the multilamellar organization of the plurethosome vesicles retained MG more efficaciously than the unilamellar structures of transethosome. However, Rv/Q6 in the case of P12-MG was almost double with respect to Sol-MG, indicating the potential of transethosome vesicle to restrain MG diffusion.

It is important to emphasize that after 120 days, P9-MG showed instability phenomena, resulting in phase separation, while in the case of P7-MG, sedimentation was detectable already after 15 days. In this regard, it is likely that the use of PC 1.8% alone could not be suitable to maintain ethosome stability, while requiring surfactants, e.g., F127 1.2 or T80 0.2%, as in the case of P11-MG and P12-MG. The different shelf life of P9-MG and P11-MG should be ascribed to the amount of F127, which in the case of P11-MG was double that of P9-MG, suggesting that the higher amount of F127 could firmer stabilize the multilamellar structure of the vesicle [44,45]. For this reason, further studies on plurethosome and transethosome were carried on using P11-MG and P12-MG.

### 3.5. Rheological Study

**Figure 5:**
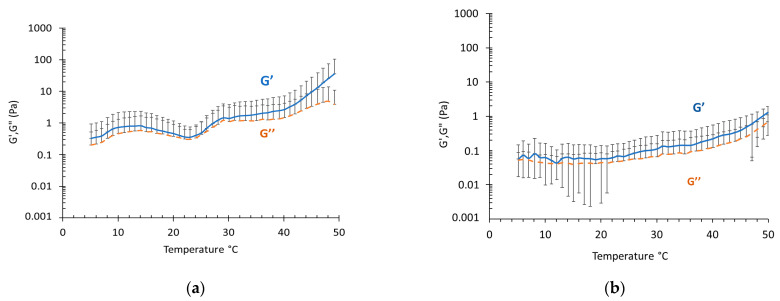
Evolution of elastic (G′, blue) and viscous (G″, orange) moduli for P11-MG (**a**) and P12-MG (**b**).

Since F127 is known to confer thermoresponsive behavior to aqueous vehicles, the rheological performance of plurethosomes was investigated. Particularly, to evaluate the role of PC and F127 on plurethosome rheology, P11-MG behavior was studied and compared to P7-MG. The elastic modulus represents the energy stored in the elastic structure of the formulation, while the viscous modulus reflects the viscous part or the dissipated energy [47,49]. Figure 5 reports the G′ and G″ moduli evolution between 5 and 50 °C, displaying low values for both samples: between ≈0.05 Pa at 5 °C and ≈1 Pa at 50 °C for P11-MG, and between 0.3 Pa at 5 °C and ≈40 Pa at 50 °C for P7-MG. The elastic modulus was slightly higher than the viscous modulus, with a minor increase upon heating. The low viscosity of plurethosomes and the absence of a thermoresponsive behavior is rather surprising, but two considerations should be made: (i) it is known that poloxamer aqueous solutions undergo thermoreversible gelation at F127 concentrations higher than 12% (*w*/*v*), whilst in a P11-MG nanosystem, F127 concentrations are 10-fold lower; (ii) the structural results by SAXS analyses suggest the positioning of F127 within the PC multilamellar vesicles, thus preventing a thickening effect of the dispersing phase [50]. Overall, G′ and G″ were higher for P7-MG than for P11-MG over the whole temperature range, which is in agreement with diffusion results indicating a higher diffusion coefficient in the case of P11-MG with respect to P7-MG. 

### 3.6. Antioxidant Activity Study

In order to investigate the antioxidant capacity of plurethosome and transethosome, the DPPH free radical scavenging activity of P11-MG and P-12-MG was compared to a MG ethanol/water solution (Sol-MG). The obtained data, reported in Table 4, agree with previous findings about the capability of ethosome and transethosome to maintain MG antioxidant potential [18]. Notably, the IC_50_ values indicated in the case of plurethosome an even higher antioxidant activity with respect to transethosome.

### 3.7. Effect of Loaded-MG against the O_3_-Induced ox-Inflammatory Damage in 3D Human Skin Models

**Figure 6:**
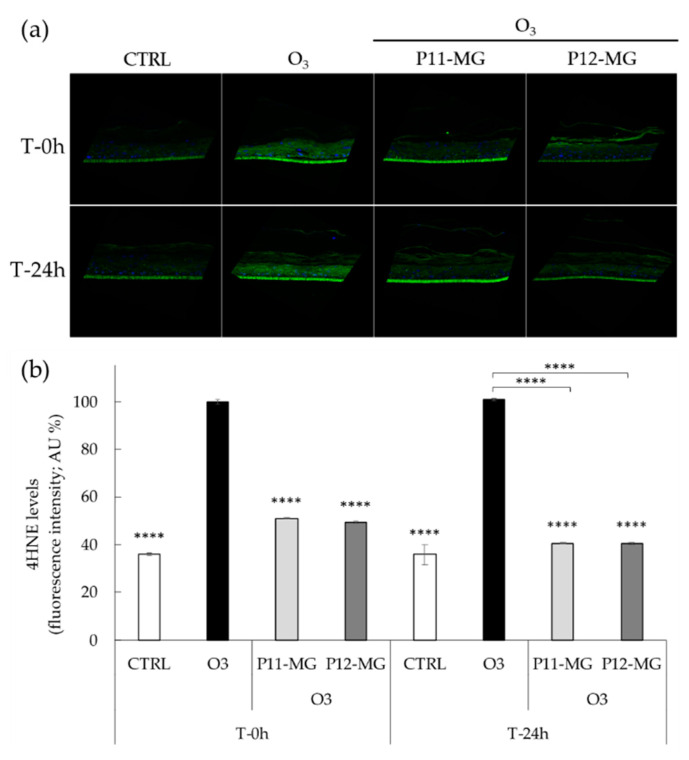
(**a**) Representative images of immunofluorescence staining for 4HNE protein adducts (green) and DAPI (blue) at 40× magnification on 3D skin models treated with P11-MG and P12-MG and exposed to O_3_ (**b**) quantification of immunofluorescence staining of 4HNE protein adducts 0 h and 24 h after O_3_ exposure. Data were normalized with respect to the O_3_-exposed sample at 0 h and expressed as arbitrary units (%) ± SD. **** *p* ≤ 0.0001 vs. O_3_ at T-0 h. The autofluorescence signal of the 3D skin model inserts was not considered in the quantification analysis.

The antioxidant and anti-inflammatory effects of MG in vitro have been recently described; particularly, the increased potential in counteracting ox-inflammatory conditions when loaded in vesicular systems has been assessed [18]. In the present study, 3D human skin models were employed to investigate the transdermal potential of P11-MG and P12-MG and their protective effect against pollutants. In this regard, the protective effect of the encapsulated MG in counteracting the cutaneous O_3_-induced oxidative stress damage was explored by evaluating 4HNE protein adducts levels, which normally occur upon O_3_ exposure and represent a marker of lipid peroxidation [51]. This reconstructed human epidermis model was selected, since it accurately resembles the human skin. Indeed, the 3D skin model is an in vitro approach widely accepted to study cutaneous drug permeability. As of 2013, reconstructed skin models received *Organization for Economic Co-operation and Development* approval for the testing of skin corrosion, acute skin irritation, and phototoxicity [52]. These models well represent skin tissues as they are characterized by both the stratum corneum and the epidermic layers, which are the most external layers of the skin. In addition, the use of these models enables preventing the variability encountered when using human skin biopsies. Particularly, 3D human skin tissues pretreated with P11-MG and P12-MG were exposed to O_3_; afterwards, 4HNE protein adducts levels were evaluated by immunofluorescence staining directly post-exposure and 24 h later (Figure 6). 

**Figure 7:**
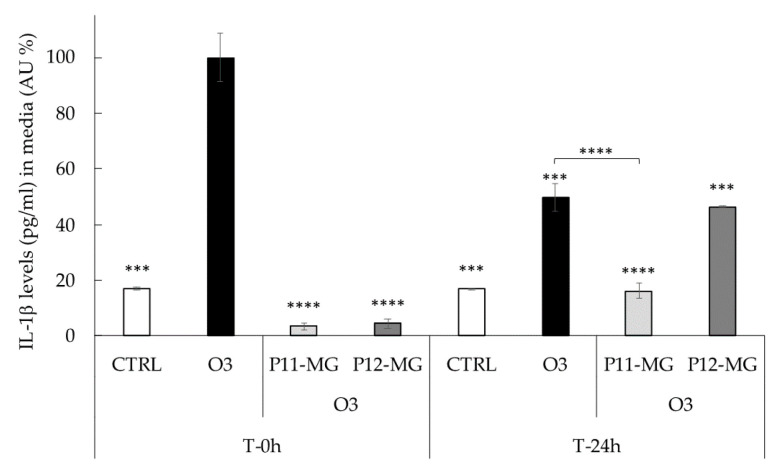
Levels of IL-1β (pg/mL) in the media of 3D skin models treated with P11-MG and P12-MG analyzed using an IL-1β ELISA kit 0 or 24 h after exposure to O_3_ 0.4 ppm for 4 h. Data were normalized with respect to the O_3_ sample at 0 h and expressed as arbitrary units (%) ± SD. Data are the results of the averages of at least three different experiments. *** *p* ≤ 0.001, **** *p* ≤ 0.0001 vs. O_3_ at T 0 h.

As depicted in Figure 6b, O_3_ exposure induced a significant increase in 4HNE protein adducts levels at both time-points (T 0 h and T 24 h) compared to untreated tissues exposed to air, while the topical administration of P11-MG and P12-MG halved the production of 4HNE levels, exerting protection against oxidative stress promoted by O_3_ exposure. Moreover, the green fluorescence intensity of P11-MG and P12-MG samples (Figure 6a) was minimal along the *stratum corneum* and epidermis, suggesting the ability of vehicles to promote MG delivery in the deeper layers of the skin. Additionally, this effect has been maintained even 24 h after O_3_ exposure, indicating the vesicle capability to retain the drug and prolong its release. Afterwards, considering the severe direct correlation between cutaneous oxidative stress and inflammation on skin upon pollutants exposure, the release of proinflammatory cytokine IL-1β was determined in 3D skin models to evaluate the inflammatory conditions upon O_3_ exposure. Figure 7 reported the analysis of IL-1β levels (pg/mL) expressed as AU% in media of 3D skin models treated with P11-MG and P12-MG. 

The high levels of IL-1β released in the O_3_ sample immediately after exposure confirmed the inflammatory status of the skin, with respect to CTRL sample. Notably, the low levels of IL-1β released, as shown in Figure 7, suggested an almost total protection against O_3_ damage exerted by P11-MG and P12-MG. Furthermore, P11-MG was even more efficacious than P12-MG in preventing the O_3_ inflammatory damage, which is still detectable after 24 h. This result suggests a longer MG anti-inflammatory effect in the case of P11-MG with respect to P12-MG, which is in agreement with in vitro diffusion data. The encouraging results suggest the potential of MG-containing plurethosome and transethosome in the treatment of ox-inflammatory damage caused by pollutants.

## 4. Conclusions

The results of this study confirmed the suitability of PC-based vesicles as transdermal delivery systems for MG. Particularly, the presence of pluronic in plurethosomes resulted in multilamellar vesicles able to firmly entrap MG and to control its diffusion more efficaciously with respect to transethosome. Three-dimensional skin models, employed to mimic the pollutant damage on human skin, confirmed that MG-containing plurethosomes were even more efficacious than transethosomes to counteract the oxinflammatory effect induced by O_3_. These encouraging results suggest the possibility to further investigate the plurethosome transdermal potential using more complex cutaneous models, such as murine models and skin explants, evaluating the presence of plurethosomes in the different skin layers, including the deeper epidermis or dermis, by transmission electron microscopy. In addition, animal models could be employed in order to study the therapeutic effect of MG loaded in plurethosome in the prevention and treatment of cutaneous conditions related to oxinflammatory mechanisms [6,7,8].

## Figures and Tables

**Table 1:** Composition of the ethosomes and plurethosomes.

Formulation Code	PC ^1^ % *w*/*w*	T80 ^2^ % *w*/*w*	F127 ^3^ % *w*/*w*	Ethanol % *w*/*w*	Water % *w*/*w*	MG ^4^ % *w*/*w*
P1	0.6	-	-	19.4	80.0	-
P2	0.6	-	0.3	19.1	80.0	-
P3	0.6	-	0.6	18.8	80.0	
P4	0.6	-	1.2	18.2	80.0	-
P5	0.9	-	-	29.1	70.0	-
P6	0.9	0.3	-	28.8	70.0	-
P7	1.8	-	-	18.2	80.0	-
P8	1.8	-	0.3	17.9	80.0	-
P9	1.8	-	0.6	17.6	80.0	-
P10	1.8	-	0.9	17.3	80.0	-
P11	1.8	-	1.2	17.0	80.0	-
P12	1.8	0.2	-	18.0	80.0	-
P13	1.8	0.2	1.2	16.8	80.0	-
P14	1.8	-	1.6	16.6	80.0	-
P15	2.7	-	-	27.3	70.0	-
P16	2.7	0.3	-	27.0	70.0	-
P7-MG	1.8	-	-	18.12	80.0	0.08
P9-MG	1.8	-	0.6	17.52	80.0	0.08
P11-MG	1.8	-	1.2	16.9	80.0	0.08
P12-MG	1.8	0.2	-	17.5	80.0	0.08

^1^: soy phosphatidylcholine; ^2^: polysorbate 80; ^3^: pluronic F127; ^4^: mangiferin.

**Table 2:** Size distribution parameters of vesicular nanosystems, as determined by PCS.

Formulation	Time	Z Average	Dispersity	Macroscopic
Code	(Days)	(nm) ± s.d.	Index ± s.d.	Appearance
P1	1	201.2 ± 1	0.06 ± 0.01	translucent
	90	206.0 ± 2	0.08 ± 0.03	translucent
P2	1	195.5 ± 1	0.14 ± 0.02	translucent
	90	200.0 ± 10	0.14 ± 0.03	translucent
P3	1	264.4 ± 14	0.15 ± 0.02	translucent
	90	n.d.	n.d.	phase separation
P4	1	243.7 ± 35	0.16 ± 0.01	milky
	90	n.d.	n.d.	phase separation
P5	1	206.3 ± 33	0.15 ± 0.04	milky
	90	225.4 ± 25	0.16 ± 0.02	milky
P6	1	186.2 ± 20	0.13 ± 0.05	translucent
	90	190.5 ± 22	0.14 ± 0.05	translucent
P7	1	285.5 ± 30	0.22 ± 0.07	milky
	90	n.d.	n.d.	phase separation
P8	1	227.7 ± 30	0.18 ± 0.05	milky
	90	n.d.	n.d.	phase separation
P9	1	237.3 ± 25	0.22 ± 0.09	milky
	90	260.2 ± 15	0.23 ± 0.07	milky
P10	1	247.8 ± 35	0.18 ± 0.05	milky
	90	270.2 ± 15	0.19 ± 0.06	milky
P11	1	252.3 ± 25	0.18 ± 0.04	milky
	90	260.2 ± 17	0.19 ± 0.08	milky
P12	1	338.5 ± 20	0.18 ± 0.07	quite translucent
	90	360.5 ± 15	0.20 ± 0.08	quite translucent
P13	1	351.2 ± 18	0.17 ± 0.06	translucent
	90	382.7 ± 15	0.19 ± 0.05	translucent
P14	1	375.6 ± 12	0.21 ± 0.09	milky
	90	n.d.	n.d.	phase separation
P15	1	320.7 ± 12	0.24 ± 0.07	milky
	90	n.d.	n.d.	phase separation
P16	1	550.5 ± 80	0.25 ± 0.05	quite translucent
	90	525.4 ± 20	0.26 ±0.07	quite translucent
P7-MG	1	274.5 ± 20	0.21 ± 0.05	yellowish
	90	n.d.	n.d.	phase separation
P9-MG	1	225.2 ± 15	0.20 ± 0.04	yellowish
	90	240.2 ± 11	0.22 ± 0.07	yellowish *
P11-MG	1	243.7 ± 25	0.18 ± 0.05	yellowish
	90	248.7 ± 16	0.17 ± 0.09	yellowish
P12-MG	1	332.5 ± 12	0.20 ± 0.04	yellowish
	90	334.0 ± 25	0.20 ± 0.08	yellowish

s.d.: standard deviation; * phase separation after 120 days; data are the mean of three independent determinations on different batches.

**Table 3:** Diffraction parameters of the indicated formulations.

Figure	Structure	Lamellar Repeat Distance (nm)
P7	multilamellar	6.76
P9	multilamellar	6.19
P11	multilamellar	6.13
P12	lamellar	-
P13	lamellar	-
P16	lamellar	-

**Table 4:** MG entrapment and DPPH radical-scavenging capacity of the indicated formulations.

Formulation	EC ^a^ (%)	Recovery ^b^ (%)	DPPH IC_50_ (µg/mL)
Sol-MG ^c^	-	-	14.06 ± 0.53
P7-MG	78 ± 2	98 ± 1	n.d.
P9-MG	87 ± 3	97 ± 3	n.d.
P11-MG	88 ± 4	99 ± 1	16.10 ± 0.23
P12-MG	71 ± 2	98 ± 2	20.89 ± 0.08

^a^: Entrapment capacity; ^b^: amount of MG associated to the vesicles plus the amount of MG found in the dispersing phase, ^c^: MG (0.8 mg/mL) in ethanol/water 30:70 (*v*/*v*).

**Table 5:** Diffusion parameters of the indicated formulations.

Parameter	P7-MG	P9-MG	P11-MG	P12-MG	Sol-MG
F ^1^ ± s.d. (μg/cm^2^/h)	26.44 ± 3.4	61.84 ± 5.2	47.36 ± 4.7	53.40 ± 5.4	73.58 ± 10.0
MG (mg/mL)	0.8	0.8	0.8	0.8	0.8
D ^2^ ± s.d. (cm/h) × 10^−3^	33.05 ± 4.2	77.30 ± 6.5	59.20 ± 5.8	66.75 ± 6.7	91.97 ± 12.5
Rm ^3^ ± s.d. (μg/cm^2^)	15 ± 4	9 ± 3	10 ± 3	11 ± 4	10 ± 2
Rv ^4^ ± s.d. (μg/mL)	610 ± 30	575 ± 45	580 ± 28	480 ± 32	360 ± 27
Q6 ^5^ ± s.d. (μg/mL)	175 ± 15	216 ± 18	210 ± 19	309 ± 28	430 ± 40
Rm/Q6	0.08	0.04	0.05	0.03	0.02
Rv/Q6	3.48	2.66	2.76	1.55	0.83

^1^: Flux; ^2^: Diffusion coefficient; ^3^: residual MG on the membrane after 6 h; ^4^: residual MG in the vehicle after 6 h; ^5^: MG diffused after 6 h; data are the mean of six independent Franz cell experiments.

## Supplementary Materials

The following are available online at https://www.mdpi.com/article/10.3390/pharmaceutics13081124/s1. Figure S1. Images and hypotetical structural organization of ethosome (a), transethosome (b) and plurethosome (c). Figure S2. TEM images of P11 (a) and P12 (b). The bar corresponds to 250 nm in panels (a) and (b) and 100 nm in the insets. Figure S3. TEM image of P 1.2% aqueous solution. The bar corresponds to 50 nm.
