# Supplementary material for: “Plurethosome” as Vesicular System for Cutaneous Administration of Mangiferin: Formulative Study and 3D Skin Tissue Evaluation
Source: Pharmaceutics. 2021 Jul 23;13(8):1124. doi: 10.3390/pharmaceutics13081124 (PMC8398752; doi:10.3390/pharmaceutics13081124)
Supplement: Supplementary file 1 [file pharmaceutics-13-01124-s001.zip › pharmaceutics-1277155-supplementary.pdf]

pharmaceutics

Supplementary Materials: "Plurethosome" as Vesicular System for Cutaneous Administration of Mangiferin: Formulative Study and 3D Skin Tissue Evaluation

Maddalena Sguizzato, Francesca Ferrara, Paolo Mariani, Alessia Pepe, Rita Cortesi, Nicolas Huang, Fanny Simelière, Paola Boldrini, Anna Baldisserotto, Giuseppe Valacchi * and Elisabetta Esposito *

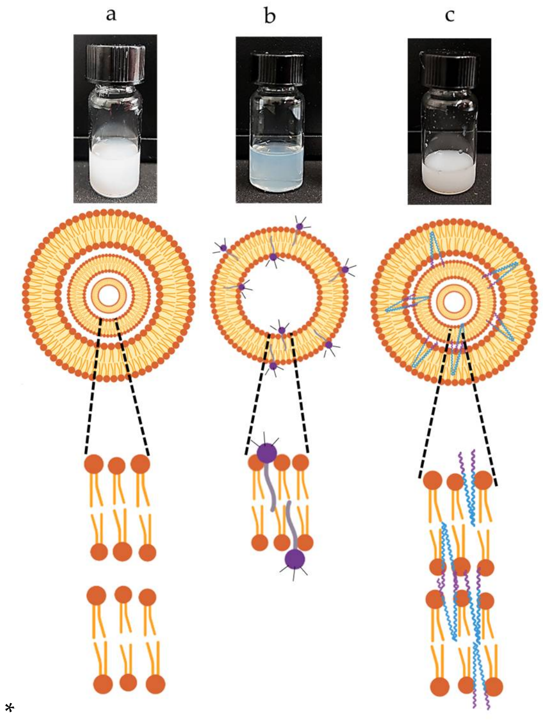

Figure S1. Images and hypotetical structural organization of ethosome (**a**), transethosome (**b**) and plurethosome (**c**).

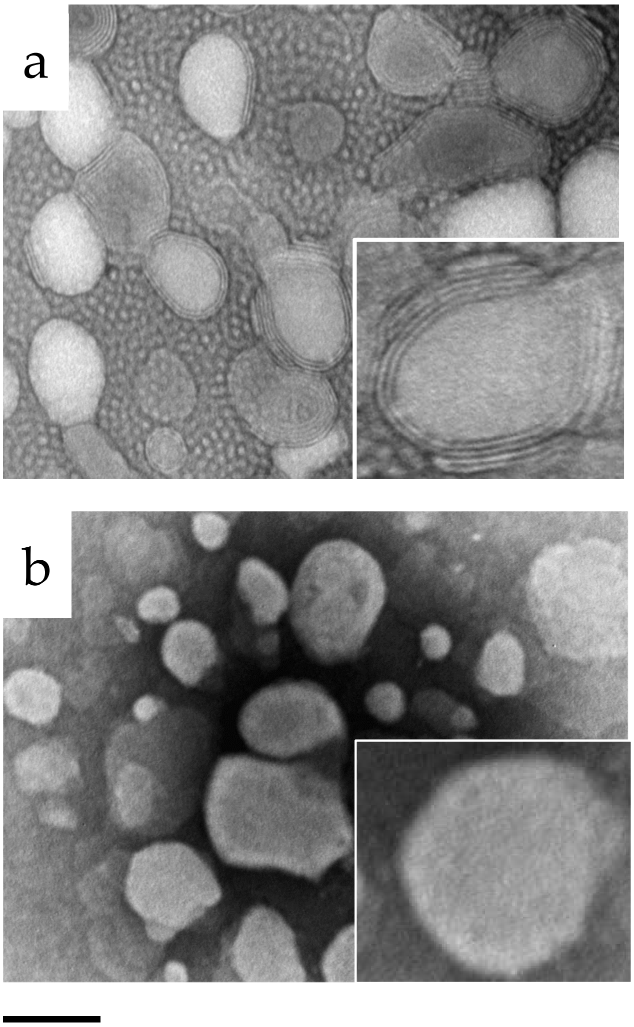

Figure S2. TEM images of P11 (**a**) and P12 (**b**). The bar corresponds to 250 nm in panels (**a**) and (**b**) and 100 nm in the insets.

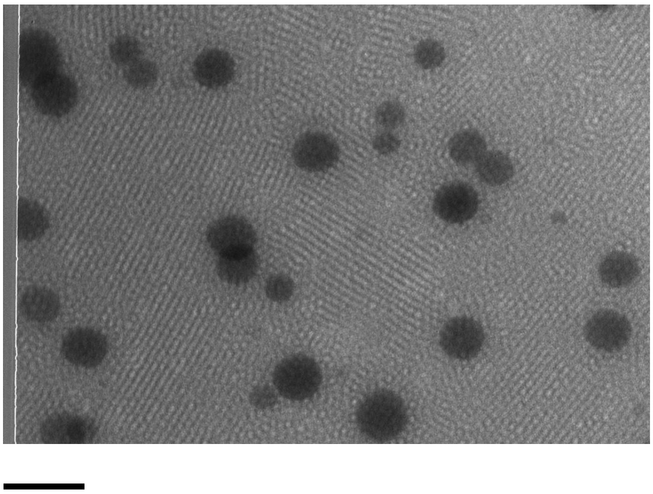

Figure S3. TEM image of P 1.2% aqueous solution. The bar corresponds to 50 nm.
